# Predictive ecological niche model for *Cinnamomumparthenoxylon* (Jack) Meisn. (Lauraceae) from Last Glacial Maximum to future in Vietnam

**DOI:** 10.3897/BDJ.12.e122325

**Published:** 2024-05-16

**Authors:** Mai-Phuong Pham, Duy Dinh Vu, Thanh Tuan Nguyen, Van Sinh Nguyen

**Affiliations:** 1 Join Vietnam–Russia Tropical Science and Technology Research Center, Hanoi, Vietnam, Ha Noi, Vietnam Join Vietnam–Russia Tropical Science and Technology Research Center, Hanoi, Vietnam Ha Noi Vietnam; 2 Graduate University of Science and Technology (GUST), Vietnam Academy of Science and Technology, Ha Noi, Vietnam Graduate University of Science and Technology (GUST), Vietnam Academy of Science and Technology Ha Noi Vietnam; 3 Vietnam National University of Forestry at Dong Nai, Dong Nai, Vietnam Vietnam National University of Forestry at Dong Nai Dong Nai Vietnam; 4 Institute of Ecology and Biological Resources, Vietnamese Academy of Science and Technologies, Hanoi, Vietnam Institute of Ecology and Biological Resources, Vietnamese Academy of Science and Technologies Hanoi Vietnam

**Keywords:** *Cinnamomumparthenoxylon*, GEE, habitat, machine learning, niche model

## Abstract

*Cinnamomumparthenoxylon* (Jack) Meisn. is a tree in genus *Cinnamomum* that has been facing global threats due to forest degradation and habitat fragmentation. Many recent studies aim to describe habitats and assess population and species genetic diversity for species conservation by expanding afforestation models for this species. Understanding their current and future potential distribution plays a major role in guiding conservation efforts. Using five modern machine-learning algorithms available on Google Earth Engine helped us evaluate suitable habitats for the species. The results revealed that Random Forest (RF) had the highest accuracy for model comparison, outperforming Support Vector Machine (SVM), Classification and Regression Trees (CART), Gradient Boosting Decision Tree (GBDT) and Maximum Entropy (MaxEnt). The results also showed that the extremely suitable ecological areas for the species are mostly distributed in northern Vietnam, followed by the North Central Coast and the Central Highlands. Elevation, Temperature Annual Range and Mean Diurnal Range were the three most important parameters affecting the potential distribution of *C.parthenoxylon*. Evaluation of the impact of climate on its distribution under different climate scenarios in the past (Last Glacial Maximum and Mid-Holocene), in the present (Worldclim) and in the future (using four climate change scenarios: ACCESS, MIROC6, EC-Earth3-Veg and MRI-ESM2-0) revealed that of *C.parthenoxylon* would likely expand to the northeast, while a large area of central Vietnam will gradually lose its adaptive capacity by 2100.

## Introduction

Global climate change and substantial illegal harvesting have been highly intricate and unpredictable phenomena, posing potential risks to human life, flora, fauna and the environment. The degradation of forest ecosystems has jeopardised the existence of various species in nature (Jhariya et al. 2019). A vivid illustration of this point is the notable decline in the population of *Cinnamomumparthenoxylon (C.parthenoxylon)*. Due to these reasons, it is imperative to conduct strategic studies across the entire territory of Vietnam to conserve this valuable plant species in nature (Nguyen et al. 2021).

*C.parthenoxylon* was initially scientifically described by Karl Friedrich Meisner (Meisn.) in 1864. This species belongs to *Cinnamomum* genus, which is naturally distributed in Cambodia; China (Guizhou, Hainan, Yunnan, Hunan, Fujian, Jiangxi, Guangdong, Sichuan, Guangxi); Indonesia (Sumatera, Kalimantan, Jawa); Lao People's Democratic Republic; Malaysia; Myanmar; Thailand; and Vietnam (IUCN). In Vietnam, it is found in provinces in the North, North Central and some Southern provinces, with a wide distribution range from 50-1500 m in elevation across various types of forests, including planted forests, production forests, natural forests and even shifting cultivation areas. The species exhibits strong regenerative capabilities (Vu et al. 2022). However, excessive exploitation is common, leading to the scarcity of natural forests in several provinces in northern Vietnam.

Trees reach maturity to 20 to 25 years of age, with a breast height diameter ranging from 30 to 35 cm and a height of 20 to 25 m (Vu et al. 2022). It grows in primary and secondary lowland to montane forests, sometimes on sands, sandstone or granite (IUCN 2022). In general, the species demonstrates ecological suitability primarily in subtropical/tropical moist forests. It is a versatile, economically valuable tree, providing high-quality timber and yielding trunk and root essential oils used in cosmetics and pharmaceuticals production, with significant export value. The seeds yield fatty acids and the essential oil has medicinal applications for treating various ailments and also misuse as a psychoactive drug (Appendino et al. 2014, Adfa et al. 2022). Excessive human exploitation has significantly impacted the size of populations (Nguyen et al. 2021).

One of the imperatives for ecologists is to identify conservation solutions for species. In the realm of biodiversity conservation, Ecological Niche Models (ENMs) have emerged as a primary method for modelling the distribution of species within a geographic area (Li et al. 2023). This constitutes an essential task for conservationists. This technique aids in identifying statistical relationships between the distribution data of a species and environmental variables. Many reforestation projects have employed ENM to identify suitable areas for cultivation, particularly for the production of forests or the cultivation of medicinal plants. Our study on the *Taccachantrieri* in a natural park of Vietnam also utilised this model to determine optimal distribution areas (Pham et al. 2023). Subsequently, the resulting seedlings, products of the breeding process, were introduced to regions with climatic conditions conducive to their development and growth. This collaborative effort involves contributions to both ecologists and the local community.

Amongst the primary types of ENMs, correlation models remain the most widely utilised in ecological and evolutionary population characteristic studies, as well as in predicting the future climate adaptation range of species populations. The correlation model assesses the potential relationships between environmental predictor factors (such as: climate data (Hijmans et al. 2005, Fick and Hijmans 2017), land-use types, soil types and topography and species spatial data (Pham et al. 2023). The widespread application of ENM techniques has facilitated researchers in generating new methods and ideas for development. Maximum entropy, developed by Phillips et al. (2006), has been particularly prevalent in ENM, with thousands of studies over the decades. MaxEnt is a machine-learning method that requires species presence data and a set of background environmental data. This work requires the use of high-configuration computers to process significant amounts of raster environmental data.

The potential applications of ENM techniques have spurred researchers to implement these methods across various platforms. For instance, the R package (Hijmans 2018) is widely used, as are comprehensive multi-model platforms such as 'SDM' (Nguyen et al. 2021) and 'biomod2' (Ngila et al. 2023). Additionally, adjustments can be made using generalised linear models to refine models, as seen with the MaxEnt approach (Phillips et al. 2017). Recently, Google developers have incorporated MaxEnt into Google Earth Engine (GEE) (Gorelick et al. 2017). GEE is a cloud-based platform for geographic spatial analysis, leveraging the processing power of Google's computational services to conduct analyses on a global to local geographical scale (Gorelick et al. 2017). Data available from GEE, such as satellite images and digital elevation models, have proven highly useful in supporting large-scale spatial modelling efforts. GEE has diverse applications in various fields, including forest management, water resource management and disease risk mapping. Many machine-learning algorithms commonly used in ENM, such as classification regression, random forests and support vector machines, have been integrated into GEE, given their frequent application in satellite image analysis. While there is still limited research conducted on ENM using GEE (Crego et al. 2022), GEE has recently demonstrated the power of cloud technology in handling large spatial data volumes, enhancing both model accuracy and run - time efficiency significantly (Amani et al. 2020).

In this study, five different machine-learning algorithms (MaxEnt, Random Forest - RF, Gradient boosting Decision Tree - GBDT, Support Vector Machine - SVM and Classification and Regression Tree – CART) were used to model the potential distribution of *C.parthenoxylon* based on data collected from 117 species occurrence points coupled with 20 environmental variables. Ultimately, we aimed to identify the most suitable machine-learning algorithms for constructing an ENM for this species through the habitat suitability index (HSI) (Crego et al. 2022). Five algorithms were employed to model the species geographical distribution in the present. Then, we selected the algorithm that provided the present model with the highest accuracy. It was applied to evaluate the future models based on climate change scenarios from different regions, including the periods 2061–2080 and 2081–2100, as well as the past models, including the Last Glacial Maximum and the Mid-Holocene. The methods in this paper can also be applied to conduct ENM studies for species within the same *Cinnamomum* genus in Vietnam.

## Materials and methods

### Species data and study areas

**Figure 1.**
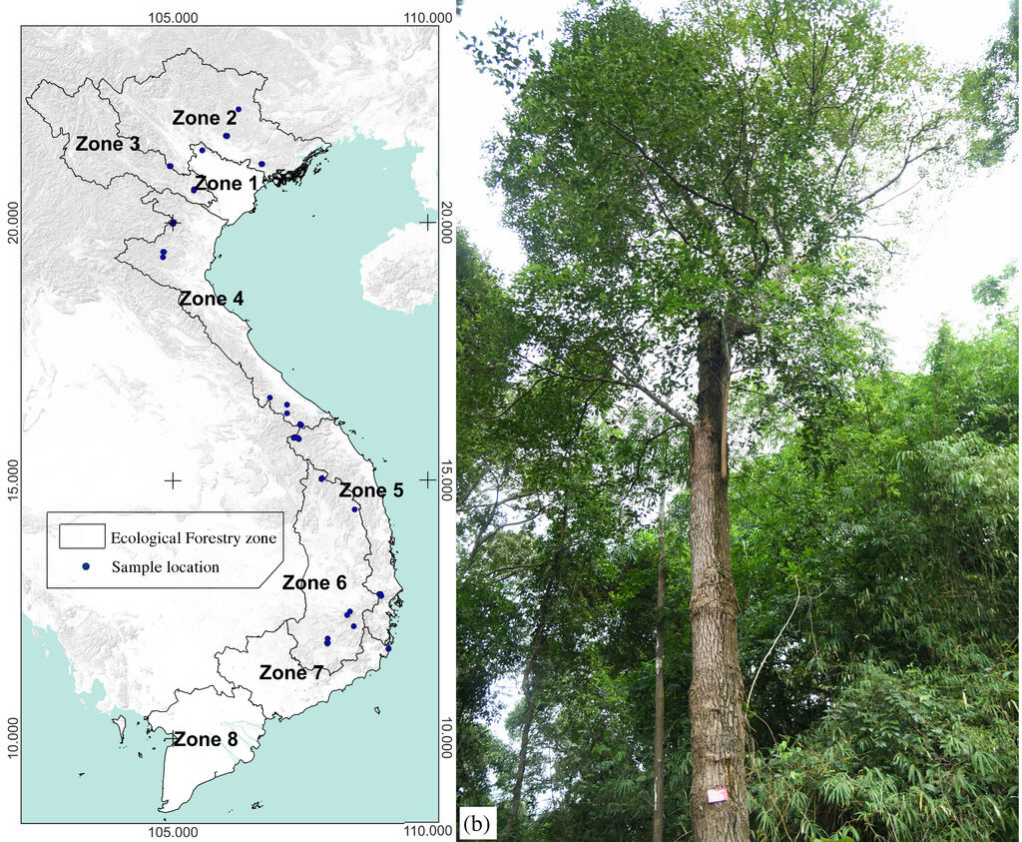
Geographical distribution of sampling points of *C.parthenoxylon* in Vietnam. Study area **(a)**; adult plant **(b)**. (**Zone 1**: Red River Delta; **Zone 2**: North East; **Zone 3**: North West; **Zone 4**: North Central Coast; **Zone 5**: South Central Coast; **Zone 6**: Central Highlands; **Zone 7**: South-East; **Zone 8**: Mekong River Delta (This map does not show offshore islands).

Understanding the characteristics of forestry ecological zones is important to the development of sustainable management strategies. This ensures that forestry exploitation is conducted in a balanced manner, minimising significant environmental and natural resource losses (Trieu et al. 2020). In this study, we investigated the species distribution across six forestry ecological zones during the period 2019–2024 to determine the habitat status and population size, including: Red River Delta (Zone 1), North-East (Zone 2), North-West (Zone 3), North Central Coast (Zone 4), South Central Coast (Zone 5) and Central Highlands (Zone 6). The authors have consulted previous scientific documents and there have been no records of the species' occurrence in habitats in South-East (Zone 7) and Mekong River Delta (Zone 8) (Fig. 1). The dataset and metadata derived from our study can be accessed on the Global Biodiversity Information Facility GBIF (https://www.gbif.org/dataset/bc8c8fdb-f776-4759-bf06-55c92e737d2b). The species' coordinate data were extracted in CSV format for use as input data in Google Earth Engine.

### Environmental parameters

We selected the environmental parameters based on their frequent applicability and ecological significance in ENM for species conservation. Finally, three sets of environmental parameters have been chosen for utilisation to predict the ENM of *C.parthenoxylon* at present, including:

- *Terrain data*: **Elevation** was used from Radar Topography Mission (SRTM) digital elevation data which is an international research effort that obtained digital elevation models on a near-global scale. This SRTM V3 product (SRTM Plus) is provided by NASA JPL at a resolution of 1 arc-second (approximately 30 m) (Farr et al. 2007).
- *Climate data*: We employed current climate data downloaded from the Worldclim ver-2.1 worldclim.org) with a resolution of 30 arc-seconds (equivalent to 1 km^2^) to determine suitable distribution areas for the species. The dataset comprises 19 climate variables, including: annual mean temperature (**bio1**); mean diurnal range (**bio2**); isothermality (**bio3**); temperature seasonality (**bio4**); max temperature of warmest month (**bio5**); min temperature of coldest month (**bio6**); annual temperature range (**bio7**); mean temperature of wettest quarter (**bio8**), mean temperature of driest quarter (**bio9**), mean temperature of warmest quarter(**bio10**) and coldest quarter (**bio11**); annual precipitation (**bio12**); precipitation of wettest month (**bio13**); precipitation of driest month (**bio14**); precipitation seasonality (**bio15**); precipitation of wettest quarter (**bio16**), precipitation of driest quarter (**bio17**), precipitation of warmest quarter (**bio18**) and precipitation of coldest quarter (**bio19**) (Fick and Hijmans 2017).

To forecast the future ENM of *C.parthenoxylon*, we utilied four climate change scenarios, including:

- **ACCESS** scenario from the Australian Research Council Centre of Excellence for Climate System Science.
- **MIROC6** scenario from the JAMSTEC (Japan Agency for Marine-Earth Science and Technology, Japan) & AORI (Atmosphere and Ocean Research Institute, The University of Tokyo, Japan) & NIES (National Institute for Environmental Studies, Japan) & R-CCS (RIKEN Center for Computational Science, Japan).
- **EC**-Earth3-Veg scenario from Swedish Meteorological and Hydrological Institute of Sweden.
- **MRI**-ESM2-0 scenario from Meteorological Research Institute of the Japan Meteorological Agency.

These scenarios were applied to models covering the periods 2061-2080 and 2081-2100. Four emission scenarios corresponding to shared socioeconomic pathways (SSP126, SSP245, SSP370 and SSP585) were considered, as provided by CMIP6 with net radiative forcing values of 2.6, 4.5, 7.0 and 8.5 W/m² (Fick and Hijmans 2017, Riahi et al. 2017), all sourced from Worldclim version 2.1, 250 m resolution.

To predict the historical ENM of *C.parthenoxylon*, we employed two paleoclimate datasets downloaded from paleoclim.org, version 1.4:

- **LGM** (Last Glacial Maximum), approximately 22,000 years ago (Karger et al. 2017),
- **MH** (Mid Holocene), approximately 6,000 years ago (Brown et al. 2018).

### Methodology

We applied five distinct machine-learning algorithms in Google Earth Engine (GEE): Random Forest - RF (Breiman 2001), Support Vector Machine - SVM (Vapnik 1995), Gradient Boosting Decision Tree - GBDT (Friedman 2001), Classification and Regression Tree - CART (Breiman 2017) and MaxEnt (Phillips et al. 2017). A total of 30% of the occurrence sample data were reserved for assessing the model's capacity, while the remaining 70% were used for training. Each run type generated a total of 10 replications, and the results were averaged. The remaining settings were maintained at their default parameters.

#### Random Forest (RF)

The Random Forest­­­ algorithm serves as an ensemble machine-learning approach applicable to both classification and regression tasks. It operates by assembling multiple decision trees during the training phase and generates outcomes in the form of mode (for classification) or average prediction (for regression) based on the individual trees (Breiman 2001). In this framework, upon providing an input to the Random Forest (RF), that input propagates to each constituent partition. Each tree autonomously predicts a classification and contributes a "vote" towards the respective class. The resulting output values are determined via the average outputs derived from all trees in the regression phase. Two essential parameters for this classification algorithm are ntree (representing the number of trees to be cultivated) and mtry (indicating the number of variables allocated for classification at each node). The selection of the sub-set of ntrees is contingent upon achieving the shortest processing time for attaining the lowest error. The ntree range spans from 1 to 1000 trees, while mtry ranges from the minimum number of variables (minimum independence being 1) to the smallest count of independent variables utilised in classification. Notably, the Random Forest algorithm has a built-in feature selection system that makes it easier to process a lot of input parameters without having to delete parameters to make the algorithm smaller.

#### Support Vector Machine (SVM)

SVM is a supervised machine-learning algorithm introduced by Vapnik (1995) for classification and regression tasks. Its primary goal is to find the best possible decision boundary that separates different classes in the data by maximising the margin between them. This optimal hyperplane maximises the distance between the nearest training samples and the separating hyperplane (Melgani and Bruzzone 2004). Due to the division of the feature space by hyperplanes, addressing non-linear layer boundary problems results in lower accuracy. Thus, SVM employs the "kernel trick" to project data into a higher-dimensional feature space (Fassnacht et al. 2014), enabling the establishment of non-linear class boundaries. Standard SVM algorithms might underperform with highly imbalanced training sample sets or mislabelled samples. This issue arises because the cost function, guiding standard SVM training, is penalised by misclassified samples.

#### Gradient Boosting Decision Tree (GBDT)

The research employed the Gradient Boosting Decision Tree (GBDT) machine-learning algorithm, a recursive decision-tree method consisting of multiple decision trees (Friedman 2001). This technique involves iteratively combining multiple trees to arrive at conclusive decisions. In contrast to logistic regression, which is limited to linear regression, GBDT demonstrates versatility in addressing various regression problems, both linear and non-linear and applies to binary classification as well. Notably, XGBoost (Extreme Gradient Boosting) stands out as an efficient implementation of GBDT due to its superior performance. It optimally combines software and hardware enhancements, delivering superior results, while utilising fewer computing resources compared to alternative methods. XGBoost utilises the "max_depth" parameter, specified upfront instead of relying on a criterion-first approach and employs a depth-first strategy for tree pruning, enhancing computational efficiency significantly. Additionally, the algorithm incorporates the distributed weighted quantile sketch technique to effectively identify optimal split points amongst weighted datasets.

#### Classification and regression tree (CART)

The CART algorithm divides the n-dimensional space into rectangles that do not overlap each other by recursion (Breiman 2017). First, an independent variable xi is selected, and then a ui value corresponds. The n-dimensional space is divided into two parts. Some points satisfy x_i_ = u_i_, while others satisfy x_i_ > u_i_. For a Variable that is not continuous, only two values are equal or not equal. During the filing process rules, these two parts are based on the first step to choose again an attribute and continue partitioning until dividing the n-dimensional space. Properties have the minimum GINI coefficient value to be used as partition index. For dataset D, coefficient *GINI* is defined as follows in Eq. (1).

GINI x (D) = 1 - \begin{varwidth}{50in}\begin{equation*}
            ?i
        \end{equation*}\end{varwidth} = *kp_i_^2^ (1)*,

in which k denotes the count of distinct sample types and pi signifies the probability of classifying a sample into type i. A lower GINI value indicates higher sample quality and improved sorting effectiveness. The decision tree comprises multiple levels of nodes and leaves. The term "maximum nodes" pertains to the highest number of leaves achievable per plant, while the "minimum leaf population" is the smallest number of nodes generated exclusively for training purposes. To construct an appropriate tree, enough nodes and branches must be generated. The maximum node value has no upper limit unless explicitly specified.

#### Maximum Entropy (MaxEnt)

Species Distribution Models (SDMs) are currently applied in various popular applications, including the modelling of bioclimatic conditions, defining environmental envelopes, conducting climate change experiments, employing genetic algorithms for rule-set production and utilising MaxEnt for shaping tissues (maximum entropy). Amongst SDMs, the MaxEnt model is prioritised due to its outstanding advantages, such as requiring only current species data as input. It accurately constructs spatial environment maps suitable for the species and assesses the importance of environmental variables in species distribution. The MaxEnt model can simultaneously incorporate both continuous and discrete variables as input data. This model has been widely used in habitat zoning for the conservation of various plant species worldwide (Phillips and Dudík 2008, Warren and Seifert 2011, Nguyen et al. 2021).

To understand the representation of the realised distribution of the species by p, we should examine the following sampling approach. An observer randomly selects a site, denoted as x, from the set X comprising sites within the study area. The observer records 1 if the species is present at x and 0 if it is absent. If we designate the response variable (presence or absence) as y, then p(x) represents the conditional probability P(x|y = 1), indicating the likelihood of the observer being at x given that the species is present. Applying Bayes' rule (2):

(P(y=1|x)=(P(x|y=1)P(y=1))/(P(x))= p(x)P(y=1)|x|) (2)

According to our sampling strategy, P(x) = 1/|X| for all x. In this context, P(y = 1) represents the overall prevalence of the species in the study area. The quantity P(y = 1|x) is the probability of the species being present at the location x, taking values of 0 or 1 for plants, but potentially ranging from 0 to 1 for vagile organisms (Phillips and Dudík 2008).

### Model evaluation analyses

The accuracy of the models is based on validation sets for each model iteration. The first metrics are the threshold-independent areas under the ROC curve (AUC-ROC). AUC-ROC ranges from 0 to 1, where 1 signifies perfect discrimination between true positive and false positive instances. Similar evaluations using AUC-ROC have been extensively detailed in the study of Crego et al. (2022) on four machine-learning algorithms.

### Habitat suitability index (HSI) evaluations

The Habitat Suitability Index (HSI) is an index that represents ENM through a digital map. The output from each model in various periods generates a Habitat Suitability Index (HSI) map. HSI is the result file in the last step on GEE. Then, it will be exported from GEE to Google Drive. Finally, the data will be imported into QGIS 3.22 for classification using a five-category habitat suitability index for *C.parthenoxylon*. Extreme suitable (HSI > 0.8), high suitable (HSI: 0.7 – 0.8), moderate suitable (HSI: 0.6 – 0.7), moderate-low suitable (HSI: 0.4 - 0.6), low or unsuitable (HSI: <0.4).

## Result

### Appropriate machine-learning algorithm for mapping the ENM of C.parthenoxylon in Vietnam

The evaluation results of the accuracy of species distribution models generated by five machine-learning algorithms for the validation dataset indicate that the Random Forest (RF) algorithm achieved the highest accuracy with an AUC-ROC value of 0.88. The following RF, GBDT, CART, MaxEnt and SVM algorithms demonstrated accuracies of 0.86, 0.82 and 0.68, respectively. Consistent with these findings, the RF algorithm also exhibited superiority over eight other machine-learning algorithms (SVM, GARP, DT, RIPPER, KNN, Logistic, ANN and NativeBayes) when constructing distribution models for plenty plant species in the Latin American Region, achieving AUC accuracies ranging from 0.82 to 0.96 (Lorena et al. 2011). In another study in Vietnam, Nguyen and Phung (2023) utilised Google Earth Engine to model *Hopeaodorata* using four machine-learning algorithms (RF, SVM, CART and GBDT), with the RF algorithm yielding the most accurate outcomes and the AUC was 0.89 (Nguyen and Phung 2023).

Random forest (RF) has emerged as a valuable methodology in the academic realm for modelling plant and animal habitats, as well as for monitoring alterations in land use, encompassing shifts in forest cover, land degradation and urban expansion. Moreover, its utility extends to the domain of natural disaster forecasting (Venkatappa et al. 2020), wherein it leverages diverse data sources including meteorological observations, satellite imagery and environmental parameters to anticipate and mitigate the adverse effects of events such as floods and wildfires. Random forest techniques are also very useful for studying climate change because they help figure out how much changes in the climate will affect ecosystems, which makes it easier to predict how the environment will change in the future. Campos et al. (2023) introduced the inaugural implementation of GEE and conducted a comprehensive evaluation of MaxEnt, the prevailing method in ecological niche modelling. The results demonstrate that GEE modelling yields high-performance ENMs and produces spatial predictions of comparable reliability to those generated by the widely adopted MaxEnt software, across various case studies.

### Current Habitat Suitability for C.parthenoxylon in Vietnam

The results obtained showed that *C.parthenoxylon* was naturally distributed in Vietnam, primarily in the northern regions, specifically Zones 2 and 3, North Central Vietnam (Zones 4 and 5) and the Central Highlands (Zone 6). According to Vu et al. (2022), *C.parthenoxylon* was widespread in evergreen broad-leaved forest states in most provinces in northern Vietnam. Therefore, this study supplements the appropriate distribution areas for the species in the provinces of North Central Vietnam and the Central Highlands.

**Figure 2.**
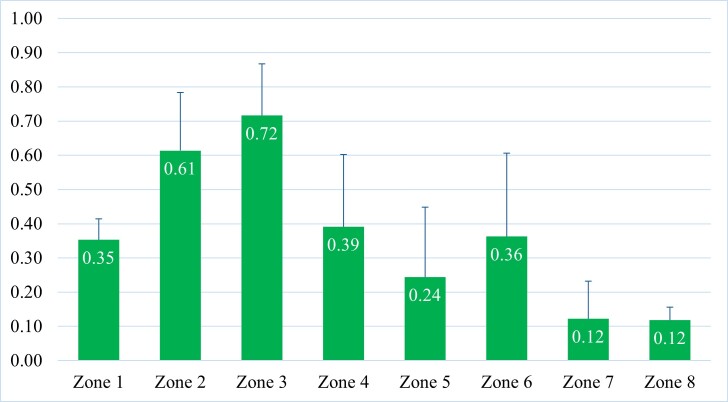
The average HSI value and standard deviation (STD) of eight Forestry Ecological Zones in Vietnam.

**Figure 3.**
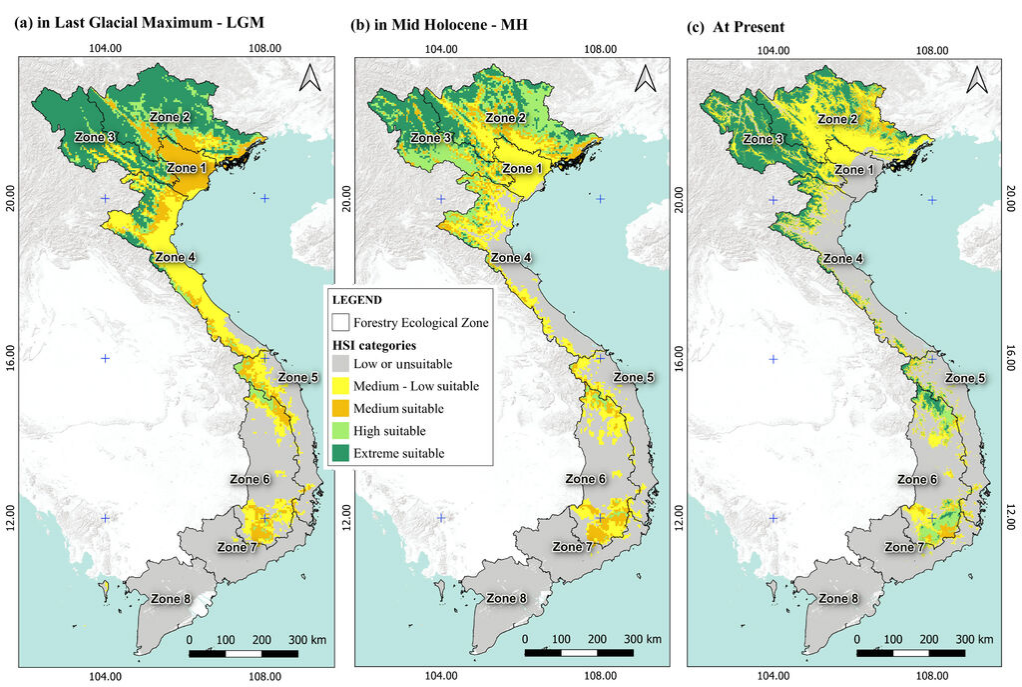
Maps of HSI for *C.parthenoxylon* under the different periods in Vietnam. LGM **(a)**; MH **(b)**; At Present **(c)**.

The simulation results using the Random Forest algorithm revealed areas classified as extremely high suitability, high suitability, medium suitability, medium-low suitability and low or unsuitable for *C.parthenoxylon*, covering 48,371.48 km^2^, 54,225.77 km^2^, 38,838.62 km^2^, 42,950.08 km^2^ and 137,384.93 km^2^, respectively. These areas correspond to 15%, 16.8%, 12%, 13.3% and 42.7% of the total area of Vietnam. Amongst them, the highest suitability area is in Zone 3, followed by Zones: 2, 4, 6, 1, 5, 7 and 8, with the average of Habitat Suitability Index (HSI) values decreasing in the following values: 0.72, 0.61, 0.39, 0.36, 0.35, 0.24, 0.12 and 0.12 (Fig. 2, Fig. 3c).

### Dynamics of Habitat Suitability area (HSI) for C.parthenoxylon under past and future scenarios

The ecologically suitable areas for species have exhibited significant fluctuations from the Last Glacial Maximum (LGM) period to the present, particularly demonstrating erratic changes in the most extremely suitable regions, notably in northern Vietnam. In this geographical area, there was a considerable loss of suitable habitat from the LGM to the Mid-Holocene (MH) period, with slight expansion from the MH period to the present (Fig. 3a, b). Notably, the emergence of the Central Highlands (Zone 6) as a new ecologically suitable zone after the MH period is evident (Fig. 3c). This shift during the transition from the mid-Holocene to the present was attributed to a gradual increase in mean annual precipitation and a reduction in both the duration and severity of the dry season.

**Figure 4.**
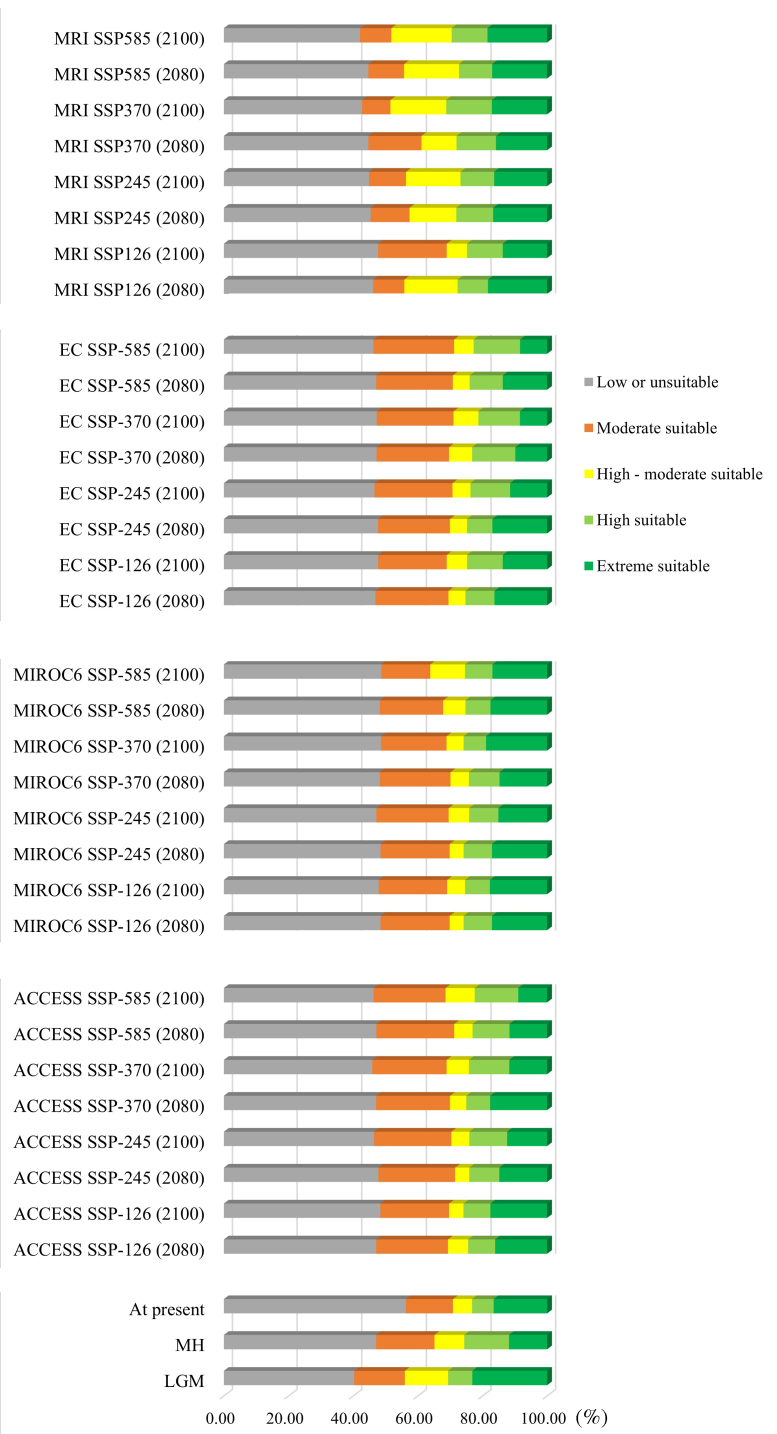
Changes in the distribution of territories, based on predictive HSI across future periods in Vietnam. Three types of data were used to prepare the 35 HSI maps: two datasets simulating past climate (LGM and MH), four datasets of future climate scenarios (ACCESS, MROC6, EC and MRI) corresponding to four emission sets (SSP 126, 245, 370 and 585) for 2080 and 2100 and one current climate dataset. This chart produces the statistics for the suitable area of this species for the environment through 35 horizontal bars representing the proportion of suitable area in the total area of Vietnam. The results of each horizontal bar are divided into four different suitability levels: extremely suitable zone (green), highly suitable zone (light blue), high-moderate zone (yellow), moderate (orange), low or unsuitable (grey).

**Figure 5.**
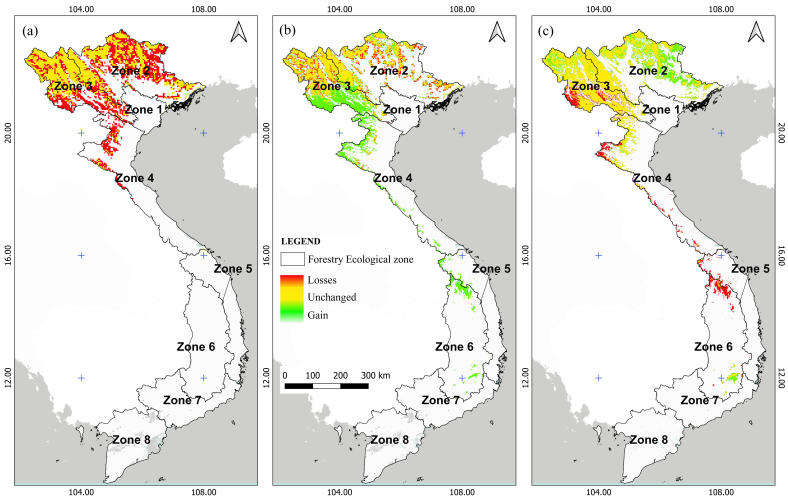
Projected changes in extremely suitable areas of *C.parthenoxylon* in Vietnam during the Mid Holocene (MH) compared with Last Glacial Maximum (LGM) **(a)**; in the present compared with Mid Holocene (MH) **(b**); in the future in agreement with ACCESS scenario, SSP-126 for 2100) compared with the present **(c)**.

The model incorporates two climate change scenarios to assess two time periods for the species distribution: 2061–2080 and 2080–2100, under the best emission scenario SSP126 and the worst emission scenario SSP585. Fig. 5 demonstrates that the ACCESS and EC scenario distinctly depict a diminishing trend in ecologically suitable areas, particularly pronounced in the Central Highlands (Zone 6) and gradually declining towards the northern regions (Zones 1, 2 and 3). Generally, Zones 3, 4, 5 and 6 are the most affected by climate change. In contrast, the dynamics of suitable area changes observed when utilising the MIROC6 and MRI scenario is insignificant (Fig. 4 and Fig. 5).

**Figure 6.**
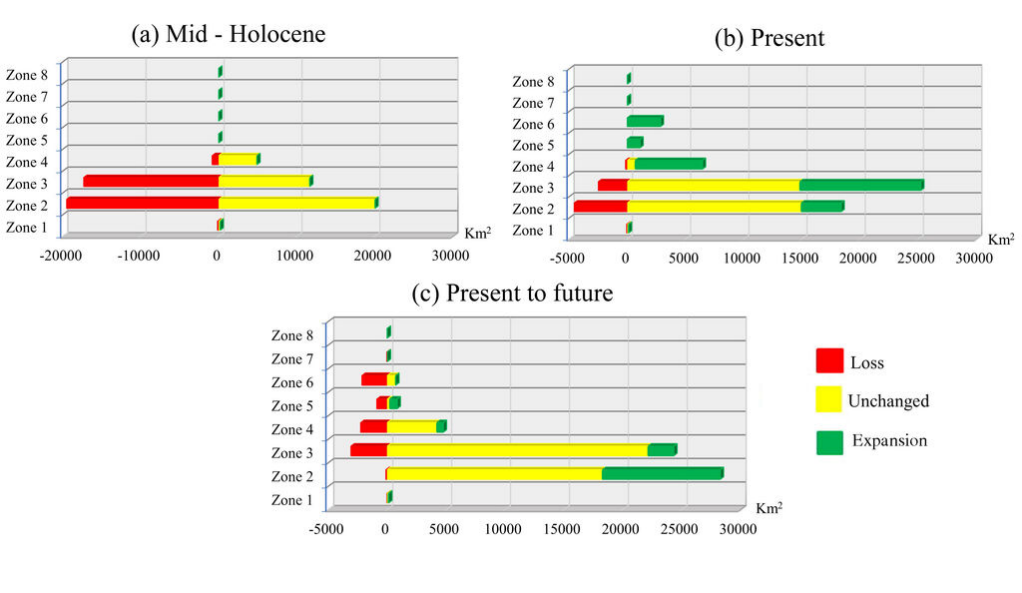
Changes in the extremely suitable areas for *C.parthenoxylon* in Vietnam during the Mid Holocene (MH) **(a)**; in the present **(b)**; in the future in agreement with ACCESS climate scenario, SSP-126 for 2100 **(c)**.

A pattern of declining suitable habitats was observed for *C.parthenoxylon* from LGM to the MH period. The total suitable area had lost nearly 50% compared to the expanded suitable area (Fig. 5a and Fig. 6a). As of the present period, the lost areas only account for one-third of the expanded areas (Fig. 5b and Fig. 6b). The most significant loss in suitable species distribution was observed in the northern regions, particularly concentrated in Zones 2 and 3. Despite increases in suitable areas across most remaining ecological zones from Zone 2 to Zone 6 over the LGM, MH and present periods, there has been no notable expansion in southern Vietnam (Zones 7 and 8).

Comparing four climate change scenarios showed that the ACCESS scenario emerged as depicting the most pronounced future decline in distribution area of *C.parthenoxylon*. Consequently, this section focuses on the separate evaluation of the ACCESS scenario for eight ecoregions in Vietnam. The findings revealed a concentration of lost suitable areas in central Vietnam across Zones 3–6, with a minor increase in suitable areas noted in Zones 2 and 3 (Fig. 5c and Fig. 6c). Broadly, a northeastward expansion trend is observed for highly suitable areas, while ecologically diminishing areas are progressively extending southward.

### Important parameters determining the distribution of C.parthenoxylon in Vietnam

**Figure 7.**
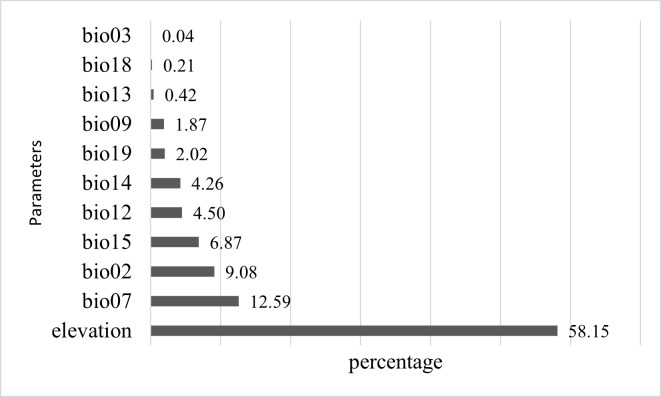
The proportion of significant parameters influencing the distribution of *C.parthenoxylon* in Vietnam. (*bio07*: annual temperature range; *bio02*: mean diurnal range; b*io15*: precipitation seasonality; *bio12*: annual precipitation; *bio14*: precipitation of driest month; *bio19*: precipitation of coldest quarter; *bio09*: mean temperature of driest quarter; *bio13*: precipitation of wettest month; *bio18*: precipitation of warmest quarter; *bio03*: isothermality).

In the forthcoming period, elevated precipitation and temperature will likely lead to an expansion of the species' suitable habitat towards the northeast, the northeast also being the most suitable habitat during the LGM period. This underscores the species' high sensitivity to various extreme climatic factors. Through an analysis of the determinants influencing species distribution, it is evident that elevation was the most important parameter influencing the distribution of *C.parthenoxylon* in Vietnam, followed by **bio07** (annual temperature range) and **bio02** (mean diurnal range) emerged as pivotal climatic variables influencing the redistribution of suitable zones, which may either expand or contract in the future (Fig. 7). These novel findings suggest that managers will be equipped with viable strategies to safeguard the species by enlarging the suitable habitat areas. Consequently, the research outcomes offer fresh insights into resource management approaches tailored to the conservation of this species.

## Discussion

### GEE is a useful tool for species conservation

Our results align well with several studies worldwide and in Vietnam, demonstrating the potential of Google Earth Engine (GEE) to provide timely and high-performance species distribution models. Moreover, the models can integrate multiple parameters available on publicly accessible cloud-based data (Crego et al. 2022). While numerous machine-learning methods have been proposed, for each environmental and species distribution dataset, we recommend employing multiple machine-learning algorithms to select the most optimal model (Nguyen and Phung 2023). The number of samples gathered to determine species distribution has a notable impact on predictions. Therefore, when working with datasets that encompass the distribution of diverse species populations, there may be variations in the selection of input parameters associated with ecological and environmental factors.

### Recommendations for species conservation of C.parthenoxylon

A primary concern in evolutionary and ecological studies involves the factors influencing and sustaining the geographic distribution of a species. This study revealed that elevation significantly influences species distribution. Additionally, variables that may explain species' climatic requirements are two temperature-related variables, namely annual temperature range *bio7* (contributing significantly at 12.56%) and mean diurnal range *bio2* (9.08%). Temperature fluctuations over the year (*bio7*) and month (*bio2*) typically represent highland and temperate climate characteristics. Previous literature has demonstrated that low temperatures negatively impact the emergence and mortality of seedlings within the genus *Cinnamomum* (Li et al. 2023). Besides temperature, some research suggests that a species' adaptation can also be affected by annual precipitation. The optimal annual precipitation for the growth of species within the genus *Cinnamomum* ranges from 900 to 2500 mm (Meng et al. 2021). In this study, Precipitation Seasonality (*bio15*) also significantly influences species distribution, accounting for 6.87% of general impact. Therefore, climate change with increased precipitation will likely expand the distribution range of the species towards the northeast. This finding aligns with the Meng et al. (2021) study, which suggests conserving a species within the genus *Cinnamomum* in areas with suitable soil moisture to mitigate the impacts of drought. Many global climate models predict that global warming will continue at a rate of 0.2°C per decade (Araujo and New 2007). The anticipated impacts in many cases suggest that the altitude and latitude of suitable habitats for many species are changing to cope with climate change at the regional scale. Meanwhile, some species may adapt physiologically or behaviorally (Singh and Kushwaha 2011). The study indicates that habitats with suitable climatic conditions for *C.parthenoxylon* are predicted to continue expanding geographically, particularly towards the north. The timing of phenological events such as blossoming period also has potential ecological consequences.

### Limitations of the study

Various factors can influence the size of an ecological niche, such as recent human activities, geographical barriers and biological interactions (parasites, predators or competitors), which may be overlooked when predicting potential geographic distributions (Meng et al. 2021). Therefore, in addition to the environmental variables utilised in our current study, other factors such as natural history, anthropogenic pressures, impacts of natural enemies on prey species or inter-species competition may affect the suitability of the habitat. To achieve this goal, predictive outcomes need to be validated, based on the natural history knowledge of the species.

In this study, our limitation is that the models were assessed for accuracy by only AUC-ROC. AUC-ROC is considered suitable for extensive research areas with abundant species data and it often provides high accuracy, even when dealing with a small and restricted sample size (Lobo et al. 2008). Therefore, future studies should additionally evaluate algorithm accuracy using AUC-PR. Sofaer et al. (2019) demonstrated that AUC-PR is not influenced by the number of absences, making it a preferred metric due to its sensitivity in accurately predicting presence locations, especially across extensive spatial domains or when modelling the distribution of rare species (Crego et al. 2022).

## Conclusions

Our main goal was to look at how to make Ecological Niche Models (ENMs) using common techniques in the Google Earth Engine (GEE) platform. We used five different machine-learning algorithms: Random Forest (RF), Support Vector Machine (SVM), Classification and Regression Trees (CART), Gradient Boosting Decision Tree (GBDT) and Maximum Entropy (MaxEnt). The outcomes revealed that RF exhibited superior predictive accuracy in comparison with another algorithms. In addition, our study looked at four different climate change scenarios: ACCESS, MIROC6, EC-Earth3-Veg and MRI-ESM2-0. These scenarios had different levels of emissions, ranging from the most optimistic (SSP-126) to the most pessimistic (SSP-585). Our findings elucidated that the ACCESS scenario delineated a discernible trajectory of diminishing potential suitable habitats within the confines of Vietnam. Notably, notwithstanding this reduction, pockets of highly suitable areas persisted and even expanded towards the north-eastern regions of the country in light of future projections.
